# Out of Control: The Role of the Ubiquitin Proteasome System in Skeletal Muscle during Inflammation

**DOI:** 10.3390/biom11091327

**Published:** 2021-09-08

**Authors:** Stefanie Haberecht-Müller, Elke Krüger, Jens Fielitz

**Affiliations:** 1 Institute of Medical Biochemistry and Molecular Biology, University Medicine Greifswald, 17475 Greifswald, Germany; Stefanie.Haberecht-Mueller@med.uni-greifswald.de; 2 DZHK (German Centre for Cardiovascular Research), Partner Site Greifswald, 17475 Greifswald, Germany; 3 Department of Internal Medicine B, Cardiology, University Medicine Greifswald, 17475 Greifswald, Germany

**Keywords:** ubiquitin-proteasome system, proteostasis in skeletal muscle, muscle wasting, ICUAW, autoinflammation

## Abstract

The majority of critically ill intensive care unit (ICU) patients with severe sepsis develop ICU-acquired weakness (ICUAW) characterized by loss of muscle mass, reduction in myofiber size and decreased muscle strength leading to persisting physical impairment. This phenotype results from a dysregulated protein homeostasis with increased protein degradation and decreased protein synthesis, eventually causing a decrease in muscle structural proteins. The ubiquitin proteasome system (UPS) is the predominant protein-degrading system in muscle that is activated during diverse muscle atrophy conditions, e.g., inflammation. The specificity of UPS-mediated protein degradation is assured by E3 ubiquitin ligases, such as atrogin-1 and MuRF1, which target structural and contractile proteins, proteins involved in energy metabolism and transcription factors for UPS-dependent degradation. Although the regulation of activity and function of E3 ubiquitin ligases in inflammation-induced muscle atrophy is well perceived, the contribution of the proteasome to muscle atrophy during inflammation is still elusive. During inflammation, a shift from standard- to immunoproteasome was described; however, to which extent this contributes to muscle wasting and whether this changes targeting of specific muscular proteins is not well described. This review summarizes the function of the main proinflammatory cytokines and acute phase response proteins and their signaling pathways in inflammation-induced muscle atrophy with a focus on UPS-mediated protein degradation in muscle during sepsis. The regulation and target-specificity of the main E3 ubiquitin ligases in muscle atrophy and their mode of action on myofibrillar proteins will be reported. The function of the standard- and immunoproteasome in inflammation-induced muscle atrophy will be described and the effects of proteasome-inhibitors as treatment strategies will be discussed.

## 1. Introduction

The skeletal muscle contributes approximately 40% of our body weight and is one of the biggest organs of our body in terms of mass and protein content. Nevertheless, the mass of skeletal muscle is highly dynamic and depends on physiological and pathological conditions such as postnatal and adolescent growth, exercise, nutrition, aging, cancer or inflammation. Physiological homeostasis of skeletal muscle is determined by anabolic and catabolic protein metabolism controlled by extracellular and intracellular signals. Protein homeostasis (proteostasis), in general, represents the critical balance of protein synthesis, quality control and degradation. In any cell, two major intracellular protein degradation systems exist: the ubiquitin proteasome system (UPS) and the autophagy-lysosomal pathway (ALP). Whereas ALP engages lysosomal proteolytic enzymes to degrade membrane proteins and protein aggregates, the UPS represents a cytosolic and nuclear machinery involved in the targeted degradation of cytosolic and nuclear proteins. Muscle contractile proteins are degraded by the UPS, the ALP, and proteases such as calpains and caspases [1]. These pathways are also coordinately activated during physiological and pathological muscle atrophy. Physiological muscle atrophy mainly occurs due to unloading, such as in athletes with training breaks, people with low physical activity, those having a sedentary life style or those with reduced gravitational load such as astronauts during space flight. It is also observed during intentional short- and long-term [2,3] head-down-tilt bed rest, used as space flight analogs to investigate mechanisms and counter measures of muscle atrophy caused by zero gravity. Pathological atrophy is caused by fasting, loss of innervation, due to neurological diseases such as stroke, and in many diseases (e.g., cancer [4], end-stage renal disease [5] and sepsis [6]). Whether or not age-related muscle atrophy can be classified as a physiological or a pathological condition warrants a detailed clinical assessment. Normal ageing of muscle is thought to begin around 25 years of age and to accelerate thereafter. Age-related muscle atrophy can be caused by numerous conditions, including a decline in physical activity, low-grade systemic inflammation, a reduction in anabolic hormones and a decrease in appetite and/or food intake [7,8]. To what extent these factors are related to normal ageing and are therefore physiological is not well defined. This situation is aggravated by a higher incidence of comorbidities and chronic medication in aged people. In light of population ageing and associated health risks, a clear distinction between physiological and pathological age-related muscle atrophy is needed.

Cellular organelles, large protein aggregates or proteins of the plasma membrane are transported to lysosomes for degradation by ALP in skeletal muscle under normal conditions as well as in response to stress stimuli [9]. The critical role of the ALP in skeletal muscle homeostasis and function is supported by the observation that several muscle diseases that are accompanied by atrophy or dystrophy show an increase in autophagosomes within myofibers promoting disruption of myofibril organization. A well-balanced autophagic flux is of great importance to prevent the loss of muscle mass since both ALP activation and inhibition exhibit a similar atrophic phenotype with only varying speed of onset [10,11]. Loss or impairment of muscle mass due to atrophy by perturbed proteostasis causes severe pathologies. Maintenance of muscle homeostasis is thus of utmost importance to human health, and the clinical burden caused by muscle wasting is high. However, our understanding of these processes is limited. One of the most devastating consequences during inflammation and critical illness is muscle wasting and long persisting weakness. The role of the UPS aside from its key E3 ubiquitin ligases is not well understood. In this review, we will address the current knowledge on the role of the proteasome in inflammation-induced muscle wasting.

### 1.1. Intensive Care Unit-Acquired Weakness (ICUAW)

Weakness and muscle atrophy have long been recognized as bystanders in patients with severe life-threatening infections and cancer. Many critically ill patients at the intensive care unit (ICU) suffer from muscle weakness. This syndrome is called intensive care unit-acquired weakness (ICUAW) and is defined as clinically detected weakness in critically ill patients where the only plausible etiology is the critical illness itself, which may persist long after ICU discharge [12]. In addition, the occurrence of ICUAW must follow the onset of critical illness to exclude other causes of weakness. The reduced muscle strength is typically observed after the acute phase of the disease, e.g., when sedation of the patients is stopped [13]. Affected patients reveal a pronounced lack of movement, which is in contrast to their level of consciousness and cooperativity. Physical examination shows diffuse, symmetric weakness involving all extremities and the diaphragm. ICUAW occurs in approximately 40% of critically ill patients [14], which prolongs ICU and hospital stay, and, due to weakness of the diaphragm and the auxiliary respiratory muscles, increases the duration of mechanical ventilation and ventilator weaning. ICUAW also increases ICU and hospital mortality [15,16,17]. Sustained weakness results in functional limitations as well as decreased employment rates and quality of life [18,19]. Post-ICU treatment, these patients often experience limitations even in simple daily activities such as getting out of bed, getting out of a chair and going up and down stairs. Muscle atrophy is the major contributor of ICUAW. Critically ill patients lose as much as 20% of muscle mass in the first 10 days of ICU stay [20]. Whereas physiological muscle atrophy or atrophy conditions under which unloading is the predominant cause are usually reversible and respond well to physiotherapy and exercise, sequelae of ICUAW may persist for years after ICU or hospital discharge [19]. The cause of ICUAW is currently unknown. A recent meta-analysis of 14 prospective cohort studies revealed that disease severity (i.e., Acute Physiology and Chronic Health Evaluation II score), female sex, sepsis, multiple organ failure, hyperglycemia, electrolyte disturbances, parenteral nutrition, medication (e.g., neuromuscular blocking agents, aminoglycoside antibiotics and norepinephrine) and long-lasting mechanical ventilation are risk factors for ICUAW [21]. For example, during ICU stay, 25% to 75% of mechanically ventilated, critically ill patients developed severe skeletal muscle atrophy and weakness [12]. Nutrition is a further important issue for critically ill patients because adequate feeding and outcome are associated with each other. Both underfeeding and overfeeding appear to be harmful to critically ill patients suggesting that caloric goals need to be correctly estimated [22]. Whether protein intake or absorption contribute to ICUAW is not well defined. Observational studies have shown that feeding higher as opposed to lower amounts of protein was associated with reductions in morbidity and mortality [22,23,24]. However, only few randomized controlled trials on enhanced protein administration are available, and those studies show only limited or no effects on functional and clinical outcomes [25,26,27]. Further studies are needed to clarify the relation between nutrition, protein intake and ICUAW. During ICU treatment, many critically ill patients have ionized calcium levels outside the reference range. These abnormal values are likely a marker of disease severity in critical illness. For example, ionized calcium values are often low in critically ill patients, especially those with sepsis, but often normalize with recovery [28,29]. Although early studies found that hypocalcemia is a risk factor for mortality in ICU patients [28,29], later studies that used more sophisticated statistical methods showed that ionized calcium concentration is not associated with hospital or ICU mortality [30]. Whether or not calcium levels should be corrected to target values warrants further investigation.

As 60–100% of patients with sepsis, which is defined as a life-threatening organ dysfunction caused by a dysregulated host response to an infection, develop ICUAW, this risk factor appears to be of utmost importance [31,32]. Sepsis is the most frequent cause of admission to an ICU and the most common cause of death in ICU [33]. Data from the Intensive Care over Nations audit (ICON) and the Sepsis Occurrence in Acutely ill Patients study (SOAP) revealed that approximately 30% of critically ill patients are either admitted with or develop sepsis during ICU treatment [34,35]. The association between infection, host response and ICUAW is supported by clinical observations obtained from critically ill patients affected with corona virus disease 2019 (COVID-19). In approximately 70% of COVID-19 patients, muscle weakness occurred during ICU treatment [36,37]. Because systemic inflammation in sepsis causes ICUAW and muscle atrophy in almost all affected patients, it is important to better understand the involved pathomechanisms to prevent or stop the disease and its progression. In this review, we mainly focus on ICUAW of skeletal muscle and the effects sepsis and inflammation have on it. How ICUAW affects the respiratory muscle and which animal models exist to investigate this pathology have been reviewed recently [38]. It is important to note that ICUAW is a multifactorial disease that cannot only be related to inflammation. Some of the most important causes of muscle atrophy, i.e., immobilization, a dysfunctioning nervous system, and inadequate nutrition have also been shown to contribute to ICUAW. Knowledge about central pathways mediating these disease mechanisms even if they have not been directly associated with ICUAW is important and will also be addressed.

Skeletal muscle is a great reservoir for amino acids that support the function of vital organs, including brain, heart and liver. Muscle breakdown may therefore protect the organism especially during the early phase of life-threatening diseases, especially sepsis. However, during sustained muscle wasting, as occurring in long-term critical illness, the negative effects of muscle atrophy and weakness will most certainly outweigh any beneficial effects of muscle protein breakdown. This also argues for an immediate initiation of therapies that aim to maintain muscle homeostasis. However, at this early time point, many patients are still mechanically ventilated and are not accessible for standard diagnostic procedures, which may delay the diagnosis of ICUAW and therefore specific therapies or even inclusion into clinical trials.

The decline in muscle mass of critically ill patients can be attributed to a reduction in the cross-sectional area of myofibers (MCSA). As shown by analyses of biopsies from the vastus lateralis muscle of ICU patients, a reduction in the MCSA of all fiber types can be found, and this reduction is more pronounced in fast twitch/type II-myofibers as compared with slow twitch/type I-myofibers. This was accompanied by a reduction in fast and slow myosin heavy chain (MyHC) protein and mRNA expression [39]. Myofiber atrophy leads to a reduction in muscle function with decreased specific force and endurance [40]. A disturbed protein homeostasis with increased degradation of structural proteins, such as MyHC, and reduced protein synthesis contribute to muscle wasting in critical illness. These disease mechanisms occur very early during the disease process [39,41,42,43], which is at least partially explained by the observation that infection-induced sepsis leads to an immediate and systemic increase in proinflammatory cytokines and acute phase response proteins. Since both proinflammatory cytokines and acute phase response proteins directly act on muscle homeostasis, early muscle pathologies are also expected.

The primary cause of muscle loss in atrophy is the strongly increased proteolysis of myofibrils, since more than 70% of muscle protein consists of myofibrillar proteins. The UPS and particularly E3 ubiquitin ligases play an important role in this process [44,45]. Myofibrils are highly organized structures consisting of thin and thick filaments that repeat in the contractile units called sarcomeres [46]. Because of that, their disassembly and breakdown is a complex and coordinated process comprising many different pathways, which will be discussed in the following paragraphs; however, we will mainly focus on the UPS.

### 1.2. The Ubiquitin Proteasome System

The majority of misfolded or otherwise damaged proteins in cells are targeted for degradation by the UPS. In addition, the abundance of myriads of regulator molecules including cyclins, transcription factors or kinases involved in signal transduction are under its control. The UPS generates peptides for major histocompatibility complex (MHC) class I antigen presentation and is thus also an integral part of our immune system [47].

**Figure 1:**
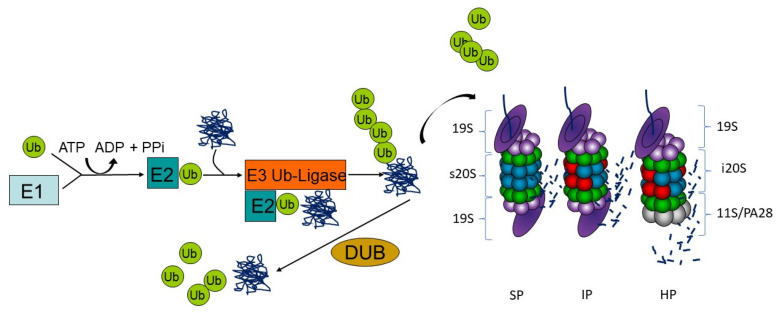
Protein substrates are covalently modified with ubiquitin by the thioester cascade. The ubiquitin-activating enzyme E1 that activates ubiquitin (Ub, green circle) under ATP-hydrolysis to form a thioester bond and transfers the activated ubiquitin to an E2 ubiquitin-conjugating enzyme. The E2 enzyme in turn interacts with its cognate E3 ubiquitin ligase to transfer the ubiquitin moiety covalently on a protein substrate. This thioester cascade has to run several times to form ubiquitin chains. Deubiquitinating enzymes (DUBs) can counteract this process to control protein degradation and recycle ubiquitin. Protein substrates modified by lysine 48 (K48)-linked ubiquitin chains are marked for degradation by different proteasome isoforms, such as standard proteasomes, immunoproteasomes or hybrid proteasomes. Immunoproteasomes contain alternative active sites, which are induced by immune signaling, whereas hybrid proteasomes additionally contain an alternative regulator complex (11S/PA28) on one site of the 20S core complex. These isoforms are thought to confer improved degradation capacities.

For degradation by proteasomes, proteins need to be post-translationally labeled with the small modifier ubiquitin. Ubiquitin modification is a sophisticated three-step enzymatic cascade involving ubiquitin activation, conjugation and ligation to a protein substrate. The ubiquitin-activating enzyme E1 activates ubiquitin in an ATP-dependent manner to form a thioester and transfers the so modified ubiquitin to an E2 ubiquitin-conjugating enzyme. The E2 enzyme in turn interacts with its cognate E3 ubiquitin ligase to transfer the ubiquitin moiety covalently on a substrate protein via an isopeptide bond. This process is run several times to generate ubiquitin chains of at least four ubiquitin moieties on substrates, which are often linked at lysine 48 (K48) of the ubiquitin amino acid sequence to be recognized by the proteasome. Substrate specificity is assured by more than 600 E3 ubiquitin ligases that are encoded in the human genome. This process can be counteracted by deubiquitinating enzymes (DUBs) that may preserve the substrate from degradation (Figure 1) [48].

Protein substrates with K48-linked poly-ubiquitin chains can be recognized for degradation by the 26S proteasome. This multi-subunit enzyme complex is generally built of two subcomplexes: the 20S proteasome core complex containing the active site subunits (standard proteasome) and the 19S regulatory complex consisting of subunits for substrate recognition, deubiquitination, unfolding and translocation into the proteolytic cavity. The attached ubiquitin is recognized by ubiquitin-binding domain proteins that function as ubiquitin receptors and decode the signal into various cellular responses [49]. Here, different ubiquitin acceptors recognize ubiquitin-modified protein substrates for degradation by proteasomes either as part of the 19S complex (subunits Rpn1, Rpn10, Rpn13 and Rpt5) or as shuttle proteins by the Dsk2, Rad23 or DDI family members [50]. The architecture of the 20S core complex is conserved from yeast to men with four staggered rings of seven subunits each. The two outer rings comprise seven different α-subunits, and the two inner rings consist of seven different β-subunits with the three active sites β1, β2 and β5 conferring the trypsin-like, the caspase-like and the chymotrypsin-like activities, respectively. Depending on cell type and tissue, proteasomes display a modular composition with isoforms that have either alternative active sites or regulator complexes or both (Figure 1). Immunoproteasomes incorporate the alternative catalytic subunits β1i (LMP2, *PSMB9*), β2i (MECL-1, *PSMB10*) and β5i (LMP7, *PSMB8*). In comparison to standard proteasomes, the assembly of immunoproteasome is significantly accelerated since β5i binds directly to the proteasome maturation protein (POMP), enabling the cell to rapidly adjust the proteolytic capacity and dynamically adapt the immune response [51]. Furthermore, the cleavage occurs more specifically after basic and hydrophobic residues, which enhances the processing of MHC class I antigens [52,53]. The altered cleavage site preference results additionally in the improvement of specific epitope amounts [54]. It was proposed that a major source for MHC class I epitopes are so-called defective ribosomal products (DRiPs), newly translated polypeptides that are immediately polyubiquitinated and allow for a very fast peptide presentation irrespective of the cellular localization of the proteins [55,56]. Nevertheless, antigenic peptides for MHC class I antigen presentation can be generated from functional proteins as well [57].

Besides that, intermediate- or mixed-type-proteasomes exist, which only contain one or two inducible catalytic subunits. They are predominantly found in tissues with high protein turnover such as tumoral tissue or the liver [58,59,60]. Different proteasome isoforms may coexist in cells and tissues. In muscle cells, standard proteasomes represent the typical proteasome population; however, the distribution of other proteasome isoforms in muscle is poorly understood.

Furthermore, the activity of the proteasome can be adjusted by association of different regulatory particles apart from 19S (PA700), namely PA28αβ, PA28γ and PA200, to one or both ends of the 20S core complex [61]. Out of those, only the activator PA28αβ is induced by proinflammatory signals and therefore often linked to immunoproteasomes in the literature, but it is found ubiquitously in all tissues [54]. Association of PA28 at one side of a proteasome complex results in so-called hybrid proteasomes (Figure 1).

Proteasome isoforms such as immunoproteasomes or hybrid proteasomes have been shown to confer altered proteolytic activities, to be involved in antigen presentation as well as in clearance of damaged proteins in the course of inflammatory signaling [47,52,62,63]. By the altered breakdown of all polyubiquitinated proteins, immunoproteasomes also affect various signaling pathways via modifying the turnover rate of regulators [47,62].

## 2. Regulation of UPS Expression by NRF1 and Other Transcription Factors

Proteasome formation occurs upon de novo synthesis via sophisticated assembly programs dependent on different signaling pathways and transcriptional programs [64,65]. Cellular requirements for proteolytic capacities can be adapted by these programs for UPS factors as well as proteasome isoforms. One of these transcriptional programs is controlled by the transcription factor nuclear factor erythroid 2-related factor 1 (NRF1), encoded by the *NFE2L1* gene, through a bidirectional regulatory feedback loop. NRF1 belongs to the Cap’n’Collar basic leucine zipper (CNC-bZIP) family and is a master regulator of adapted standard proteasome gene expression upon proteasome impairment as well as the antioxidant response and proteostasis in mammalian cells [52,66]. This so-called bounce back response of proteasomal gene activation following diminished proteasome activity is a convergent evolutionary strategy with RPN4 in yeast [67], SKN-1A in *C. elegans* [68] and Cnc-C in drosophila [69]. The two best described isoforms of NRF1 are human TCF11 (772 amino acids) and NRF1a in mice missing one exon (742 amino acids). Similar to all CNC-bZIP transcription factors, NRF1 forms dimeric complexes predominantly with small Maf (musculoaponeurotic fibrosarcoma) proteins and binds to antioxidant-response elements (ARE) in the promoter regions of its cytoprotective target genes [70]. Deletion of Nrf1 in mice is embryonic lethal due to defects in erythropoiesis and fetal liver hematopoiesis [71].

With its hydrophobic N-terminal domain (NTD), TCF11/NRF1 is tethered to the membrane of the endoplasmic reticulum (ER) [72,73]. The bulk of TCF11/NRF1 including the C-terminal DNA-binding site is located in the ER lumen [74], and some of the luminal domains are extensively N-glycosylated [75]. Due to its permanent removal by the ER-associated degradation (ERAD) system, TCF11/NRF1 has a short half-life under unstressed conditions [76]. Here, it is ubiquitinated by the ER-associated E3 ubiquitin ligase HMG-CoA (β-Hydroxy β-methylglutaryl-CoA) reductase degradation protein 1 (HRD1), extracted from the membrane by the AAA ATPase (ATPases associated with diverse cellular activities) p97/valosin containing protein (VCP) and translocated to the cytosol, where it is finally degraded by proteasomes [74,76]. When the proteasomal activity is impaired or when cells face other proteotoxic insults leading to oxidative stress [77], TCF11/NRF1 is stabilized and processed to its active form, which translocates to the nucleus and increases the expression of its target genes. Therefore, it is deglycosylated by N-glycanase 1 (NGLY1) in the cytosol [78,79]. For a long time, it was unclear which protease was responsible for the subsequent TCF11/NRF1 cleavage and activation, with calpain-1 and the proteasome being the most discussed and promising candidates [80,81]. However, Vangala and colleagues demonstrated that TCF11/NRF1 can be activated independently from proteasome activity [82], and we showed that calpain-1/2 is involved in TCF11/NRF1 degradation, but not activation under standard conditions [83]. The puzzle was solved when the aspartic protease DNA-damage inducible 1 homolog 2 (DDI2) was found to cleave TCF11/NRF1 between Trp-103 and Leu-104 at the N-terminal transmembrane domain (TMD) [74,84]. By now, it is also known that polyubiquitination is a prerequisite for proteolytic cleavage to define DDI2 as an ubiquitin-directed endoprotease [85,86].

Two other CNC-bZIP transcription factors closely related to NRF1 are NRF2 and NRF3. NRF2 is a master regulator of the antioxidative response that plays a key role in the maintenance of cellular redox homeostasis. Although both proteins have unique biological functions, NRF2 has also been shown to induce the expression of several proteasomal subunits, for example, in mice treated with high doses of antioxidants [87] or in colon cancer cells [88]. However, it is well known that NRF1 is the main regulator of adapted proteasome expression in response to proteasome impairment. In denervation-induced muscle atrophy in mice, an increased amount and activity of all catalytic standard proteasome subunits was observed, which can be attributed to elevated NRF1 protein levels in muscle tissue lysates [89,90,91].

NRF3 function has been implicated in tumorigenesis and cancer malignancy by upregulation of POMP and proteasome assembly. NRF1 and NRF3 bind complementarily to promoters of proteasome genes; however, NRF3 can suppress translation of NRF1 by the NRF3-CPEB3-NRF1 translational repression axis (for review, see [92]).

Besides the three discussed NRF transcription factor family members, other transcription factors were reported to control constitutive proteasome gene expression including nuclear transcription factor Y (NF-Y) [93], forkhead box class O family members of transcription factors (FOXO 1-4) [94,95] and signal transducer and activator of transcription 3 (STAT3) [96]. However, those transcription factors regulate the expression of only a specific set of proteasome subunits and depend on the cellular condition and the cell type [97]. Since all proteasomal subunits except RPN10 (PSMD4) are thought to only exist within their respective complex in cells and unassembled subunits are degraded, it is not clear how the upregulation of single subunits contributes to the number of complete proteasomes. These data indicate that subunit homeostasis is eventually sensed by transcriptional control circuits, as described above [92,98].

Apart from transcriptional regulation, proteasome abundance is regulated by autophagy in dependence of carbon and nitrogen sources most likely via mTOR and NRF1 [99]. However, the current knowledge about these regulatory mechanisms is limited and more studies have to be conducted in the future.

Immunoproteasomes are commonly expressed in cells of the immune system, but they can also be induced in non-immune cells upon exposure to proinflammatory cytokines such as interferons (IFN) [100] or tumor necrosis factor (TNF) [101,102], environmental stress (e.g., heat shock [103]), aging [104] or neurodegenerative diseases [105,106]. Additionally, exogenous IFN-α and IFN-β as well as endogenous virus-induced type I IFNs were also shown to increase immunoproteasome formation in cells and in vivo [107,108]. The genes of β1i (*PSMB9*) and β5i (*PSMB8*) are encoded within the MHC class II region and are continuously activated in immune cells by binding of a STAT1-IRF1-dimer to their promoter regions [60,109]. By the accelerated breakdown of polyubiquitinated proteins, immunoproteasomes may in turn affect various signaling pathways via modifying the turnover rate of regulators [47,62].

## 3. Protein Turnover in Muscle Is Regulated by a Precisely Acting Protein Degradation Pathway

**Figure 2:**
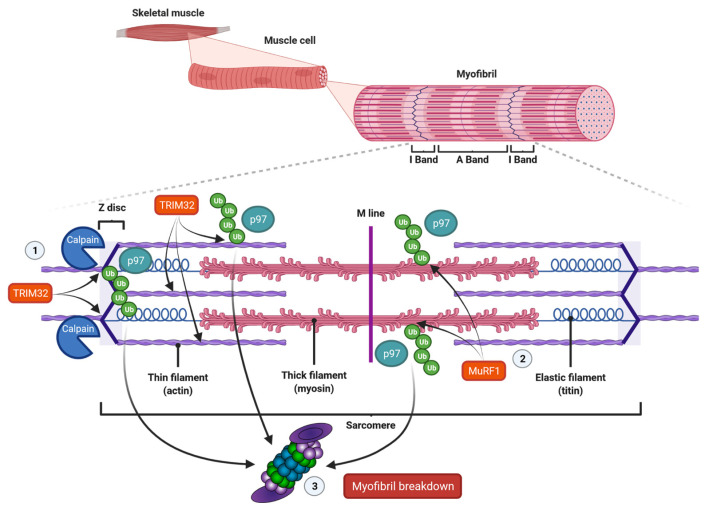
The loss of myofibrils is a highly ordered process. In a first step, calpains cleave desmin intermediate filaments (IF) to increase the accessibility for following breakdown. The E3 ubiquitin ligase TRIM32 acts on desmin IF and destabilizes the Z-disc (1). Besides that, TRIM32 is also responsible for ubiquitination of thin filaments. Next, the E3 ubiquitin ligase MuRF1 catalyzes the disassembly of thick filaments (2). Finally, the AAA ATPase p97/VCP releases ubiquitinated myofibrillar proteins to the cytosol where they are degraded by proteasomes (3). Created with https://biorender.com/.

In vertebrates, skeletal muscles are attached to bones by tendons, and they assure motion of body parts relative to each other, stability and protection of internal organs. Skeletal muscles are composed of bundled long multinuclear myocytes that contain a precisely aligned filamentous system called myofibrils. These myofibrils are responsible for force generation [110]. The smallest functional unit in myofibrils is the sarcomere, which is a repetitive unit between two Z-lines. It mainly contains thick filaments, such as myosin, and thin filaments, such as actin (Figure 2). Myosin is composed of two heavy chains (MyHC) and two pairs of regulatory (MyLC2) and essential (MyLC1) light chains, and contains an N-terminal head domain, the neck domain and a C-terminal α-helical tail domain. The myosin head contains the motor domain that binds actin and hydrolyzes adenosine triphosphate (ATP) to generate force, and its neck domain links the head and tail domains and facilitates binding with MyLCs to MyHC [110]. At the center of the sarcomere, additional structural and regulatory proteins, such as MyLCs, and myosin-binding protein C (MyBP-C), which periodically bind to the thick filaments and are required for myofibril stability and normal contractility [111,112], stabilize myosin thick filaments. The thin filaments actin, titin and nebulin are anchored to the sarcomere at the Z-band. The protein tropomyosin covers the myosin-binding sites of actin. The myosin head can only bind to actin when tropomyosin is removed from its binding site. This is realized by troponin that is aligned at intervals along the actin filaments. Troponin is composed of the three components troponin-T, troponin-I and troponin-C, and represents the on-off switch of muscle contraction [110]. Muscle contraction is activated by calcium-influx into the cytosol. Calcium ions in turn bind troponin-C and cause displacement of troponin-T and troponin-I from tropomyosin, which exposes myosin-binding sites on actin. When myosin heads bind to actin, ATP is hydrolyzed causing cross-bridge cycling with sliding of the thick over the thin filaments. This causes shortening of the sarcomeres and therefore myofibers, which results in movement. The thin filaments bind desmin via cross-linking proteins, such as α-actinin at the Z-bands. The muscle-specific type III intermediate filament (IF) desmin is essential for a proper muscular structure and function. Because desmin IF connects adjacent myofibrils with each other as well as to the sarcolemma, nucleus and mitochondria, they are important for the mechanical and structural integrity of myofibrils [113]. Importantly, it has been shown that the loss of desmin IF during starvation- or denervation-induced atrophy precedes and promotes myofibril destruction [114].

### 3.1. The Role of Calpains and the UPS in Muscle Protein Degradation

The complex structure and function of myofibrils implies that pathways involved in sarcomere disassembly and degradation are equally complicated. Because myofiber atrophy occurs while the muscle is still contracting, it appears important for the organism that the muscle keeps its functionality. Therefore, the process of myofiber degradation needs to be tightly controlled. Groundbreaking work from the groups of Goldberg, Glass and Cohen has shown that muscle atrophy is a very well-organized step-wise process. Solomon and Goldberg had already shown in 1996 that the proteasome efficiently degrades monomeric myosin, actin, troponin and tropomyosin, but that it does not break down actomyosin complexes or intact myofibrils. This observation implicates that proteins contained in multiprotein complexes or myofibrils need to be disassembled first before they can be targeted by the proteasome [44]. This disassembly is realized by proteases such as calpains or caspases that have been demonstrated to accelerate the disassembly of myofibrills [115,116]. This in turn allows proteases, E3 ubiquitin ligases and the proteasome to access monomeric actin, myosin and other associated proteins [117] (Figure 2). Although calpain [118] or caspase-3 [116] are thought to initiate myofibril breakdown, this assumption has not been proven in vivo, to date. Since the calpain- and caspase-system interact with each other, it is likely that both systems cooperate in muscle atrophy and that the impact of the one or the other may differ in individual conditions [119]. Several studies implicate that these proteases predominantly degrade desmin filaments and that the subsequent myofibril breakdown is mediated by the UPS [117]. For example, in vitro studies revealed that calpain-1 specifically cleaves isolated desmin, but not actin or myosin. Once phosphorylated and ubiquitinated, desmin is preferentially and more rapidly degraded. However, since calpain-1 does not contain a ubiquitin-binding domain, this is probably due to conformational changes [114,117,120]. Besides that, glycogen synthase kinase-3β (GSK-3β)-mediated desmin phosphorylation was shown to be required for its calpain-1-mediated depolymerization, and the subsequent myofibril destruction. Accordingly, GSK-3β inhibition in mice prevented desmin phosphorylation and depolymerization, and blocked atrophy upon fasting or denervation. GSK-3β activation is therefore an early step in muscle atrophy [120]. However, whether GSK-3β plays a role in ICUAW has not been reported.

Calpains are non-lysosomal cysteine proteases activated by calcium binding. Almost sixty years ago, they were first isolated from rat brains [121]. Besides the two ubiquitously expressed isoforms of calpain proteases, μ- and m-calpain (or calpain-1 and -2), the calpain proteolytic system comprises the small regulatory subunit CAPNS1 and the endogenous inhibitor calpastatin [122]. However, 15 other calpain isoforms with multiple splice variants and different tissue specificity exist [123]. Calpain-3 (CAPN3) is specifically expressed in skeletal muscle, and its dysfunction is linked to limb-girdle muscular dystrophy type 2A [124,125]. In addition, several studies revealed that calpain-3 levels are decreased in different atrophy conditions suggesting a unique or even contrary role of calpain-3 as compared to that of calpain-1/2, whose expression is increased in various atrophy conditions [119,126]. Calpains are modulatory proteases with little cleavage sequence specificity and limited proteolysis of their substrates; therefore, they are thought to mainly fulfill regulatory functions in physiological and pathological cellular processes [127,128]. The function of calpains in muscle wasting remains controversial. Shenkman et al. observed that inhibition of calpain-1 leads to muscle sparing and reduced protein ubiquitination in unloading-induced muscle atrophy in rats [129]. Similar observations were made after downregulation of calpain-1 by shRNA in fasting- or denervation-induced muscle atrophy, which also attenuates desmin loss and myofibril breakdown, but results in accumulation of phosphorylated desmin IF [120]. Ex vivo experiments using rat diaphragm muscle revealed that activation of calpain increased the total protein degradation rate, which was prevented by proteasome inhibition. Besides that, calpain activation suppresses the Akt pathway that promotes protein synthesis and inhibits protein degradation, probably due to proteolysis of the chaperone heat shock protein 90 (HSP 90), which enables normal Akt function. This might lead to increased expression of UPS factors in particular E3 ubiquitin ligases [130]. On the other hand, Fareed and colleagues reported that calpain inhibition also prevented sepsis- and glucocorticoid-induced muscle wasting, but did not decrease proteasomal degradation of myofibrillar proteins during sepsis. In addition, total proteasomal protein degradation and overall proteasomal activity as well as MuRF1 and atrogin-1/Muscle Atrophy F-box (MAFbx encoded by *Fbxo32*) mRNA expression were not reduced in response to calpain inhibition. These data indicate that calpain activity is not required for proteasomal degradation of muscular proteins, and the UPS is regulated independently [131].

The most important modulator of calpain activity is the amount of calcium ions. Besides that, calcium regulates the binding of the calpain inhibitor calpastatin, and active calpain degrades itself [122]. Numerous studies suggest an indirect linkage between increased intracellular calcium levels and diseases and conditions that cause muscle wasting [132]. Treatment of muscles ex vivo and myotubes in vitro with calcium or the calcium ionophore A23187 was described to increase proteasomal activity and protein degradation [133,134,135]. Notably, the depolymerization of desmin IF catalyzed by calpain-1 occurs several days after the induction of atrophy, when cytosolic calcium levels rise [120,136].

In conclusion, calpains act upstream of proteasome-dependent protein degradation during muscle atrophy. Whether or not the proteasome isoforms differentially contribute to myofibrillar protein degradation is currently unknown.

### 3.2. E3 Ubiquitin Ligases and Other UPS Factors in Skeletal Muscle

In order to identify universal markers of atrophy, two genes encoding E3 ubiquitin ligases with restricted expression to striated muscles were found to be significantly upregulated in multiple early stage models of skeletal muscle atrophy: Muscle RING (really interesting new gene) finger 1 (MuRF1 encoded by *Trim63*) and atrogin-1/MAFbx [137,138,139,140,141]. Deletion of those genes in mice partially attenuated muscle loss in denervation-induced atrophy [137]. Interestingly, Baehr et al. reported that the loss of muscle mass caused by 14 days of administration of the synthetic glucocorticoid (GC) dexamethasone is reduced in MuRF1, but not atrogin-1/MAFbx knockout mice indicative for a distinct role of both E3 ubiquitin ligases in different atrophy conditions. Since the observed partial atrophy resistance in MuRF1-deficient mice was connected to the maintenance of protein synthesis and not increasing protein degradation, another role of MuRF1 beyond the UPS is likely [142]. Furthermore, deletion of MuRF1 promotes the preservation of muscle mass in conditions of amino acid starvation [143], hindlimb unloading [144] and cardiac cachexia [145] in mice. To date, at least 120 genes named “atrogenes” have been identified with altered expression in various muscle-wasting conditions [45,141]. However, since the majority of studies employed rodent atrophy models, further investigation of the atrogene transcriptional program in human diseases is necessary.

As mentioned, myofibrillar disassembly appears to be important for proteasome-mediated degradation of structural proteins. However, since E3 ubiquitin ligases have been shown to be localized to the sarcomere, the ubiquitination of myofibrillar proteins is likely to occur independently of their disassembly. For example, MuRF1 and MuRF2 interact with the C-terminus of titin leading to their association with the M-band [146,147]. MuRF1 and MuRF3 have been shown to be localized to the Z-band [146,148]. In addition, upon denervation and fasting, MuRF1 interacts with and ubiquitinates various structural and contractile proteins such as MyBP-C, MLC1 and MLC2, even when they are organized in myofibrils, eventually leading to disassembly of thick filaments [149,150]. MyHC is also degraded in an MuRF1-dependent manner [151], but is protected in myofibrils by connected proteins [149]. These findings further support the fact that E3 ubiquitin ligases can ubiquitinate proteins contained in intact myofibrils and that the loss of myofibrillar proteins is a highly ordered process (Figure 2).

Another MuRF1 target discovered in vitro and in vivo in cultured cardiomyocytes is cardiac troponin I [152]. MuRF1 was also found to interact with titin and nebulin, and it may modulate the titin kinase activity [146,150]. Since experiments with wildtype and MuRF1 knockout mice revealed similar ubiquitination levels of some myofibrillar proteins such as titin and nebulin, it was hypothesized that they are not primary MuRF1 targets in vivo, suggesting the involvement of other E3 ubiquitin ligases [150]. In this regard, it is important to know that MuRF1 and MuRF3 physically interact with each other [146,153] and that their combined absence causes accumulations of fast- and slow-twitch MyHC in striated muscles leading to a myosin storage myopathy of the heart and skeletal muscle [151]. Likewise, the combined absence of MuRF2 and MuRF3 also leads to a protein storage myopathy in mice, in vivo [153]. However, the composition of these protein aggregates is less well defined. Nevertheless, these results imply that the MuRF family of E3 ubiquitin ligases function cooperatively and may have similar targets. Several studies showed that MuRF1 and possibly MuRF2, but not MuRF3, are linked to muscle atrophy [145,154]. A MuRF1 yeast two-hybrid screen performed by Witt and colleagues identified 11 interacting enzymes involved in ATP generation, pointing to a role as regulator of energy metabolism [150]. Beyond that, experiments with mice overexpressing MuRF1 specifically in skeletal muscle uncovered its interaction with enzymes involved in glycolysis and glycogen metabolism [155].

MuRF1 was long thought to act as a monomeric RING E3 ubiquitin ligase, but current work suggests that it may function as a component of a Cullin-type ubiquitin ligase encompassing Cullin 4A (Cul4A), DDB1, Rbx1 and DCAF8 [156]. Cullin ubiquitin ligases represent modular and versatile multi-protein complexes that are built around one of several Cullin proteins. These scaffolds bind a RING-type ubiquitin ligase and different substrate receptors, which integrate the protein complexes into diverse cellular processes [157]. In Cul4-type ligases, DDB1 and selected “DDB1-Associated Factor” (DCAF) proteins form such substrate recruiting modules [158]. Similar to MuRF1, the knockdown of DCAF8 prevented atrophy of cultured C2C12 myotubes. This finding and results from protein–protein interaction studies imply that MuRF1 and DCAF8 form an operational unit in a Cul4A ligase complex that drives myocyte atrophy [156]. Still, the detailed functions of the Cul4A-MuRF1-DCAF8-DDB1 complex in muscle biology in vivo warrant further studies.

In contrast to MuRF1, the identified substrates of atrogin-1/MAFbx are predominantly regulators of muscle protein synthesis and regeneration. The most prominent targets are the myogenic factor MyoD1 and eukaryotic translation initiation factor 3 subunit f (eIF3-f) [159,160]. Additionally, atrogin-1/MAFbx was found to interact with the desmin and vimentin IF, sarcomeric proteins and a number of other proteins in coimmunoprecipitation experiments of C2C12 myocytes treated with human myostatin, an inhibitor of myogenesis [161]. However, the interactions still need to be confirmed in vivo [154].

Whether all the identified atrogin-1/MAFbx and MuRF1-interacting proteins are also ubiquitinated by them and whether this leads to UPS-dependent degradation warrants further investigation. It is also not known which of the atrogin-1/MAFbx and MuRF1-interacting proteins are ubiquitinated and degraded during sepsis-induced muscle atrophy, and whether these substrates change with different atrophy causing conditions.

Various pathologies in and even treatments of critically ill patients can increase the activity of the UPS [162]. Importantly, an increased atrogin-1/MAFbx and MuRF1 mRNA and protein expression as well as an increased expression of the 20S proteasome was found in vastus lateralis biopsy specimens from critically ill patients at risk of developing ICUAW [39,163,164]. However, the literature is not consistent in this regard as the ubiquitin ligases are not always found to be differentially expressed in the muscle of critically ill patients [165]. The discrepancies between these studies may be related to their experimental design, timing and site of biopsy collection, and the type of analysis performed. For example, atrogin-1/MAFbx and MuRF1 are rapidly increased during atrophy conditions, which levels off in the later disease phases [39,166]. Therefore, timing of analyses is important to uncover differences in gene expression and protein contents of atrophy-related pathways; for human patients, this is not at all trivial.

### 3.3. Tripartite Motif Containing 32 (Trim32)

The E3 ubiquitin ligase tripartite motif containing (Trim) 32 is a ubiquitously expressed multifunctional protein that plays a role in differentiation, tumor suppression, muscle physiology and regeneration [167]. Similar to MuRF1, it belongs to the TRIM family of proteins, which contain three zinc binding domains composed of a RING-finger, a coiled-coil region and a B-box type 1 and 2 [168]. In contrast to MuRF1 and atrogin-1/MAFbx, TRIM32 is found in many different cell types throughout the body and is not restricted to striated muscles [154]. TRIM32 directly acts on cytoskeletal networks and insoluble myofibrils in vitro. In this regard, TRIM32 was shown to be critically linked to the breakdown of desmin IF during fasting-induced muscle atrophy, which is initiated by phosphorylation of the desmin head domain by GSK3-β or maybe other kinases, initiating the destruction of Z-bands and thin filaments [114,120]. Downregulation of TRIM32 attenuated fasting-induced breakdown of contractile and cytoskeletal proteins and subsequent muscle atrophy in hindlimb muscles. Furthermore, downregulation of TRIM32 in skeletal muscle increased PI3K/Akt/FoxO signaling, enhanced glucose uptake and induced myofiber growth [169]. The importance of TRIM32 for muscle homeostasis is supported by data from human patients. For example, *TRIM32* mutations were shown to cause limb-girdle muscular dystrophy type 2H (LGMD2H) [170]. TRIM32 was also shown to be increased in skeletal muscle of patients with Becker and Duchenne muscular dystrophy [171], whereas the latter describes a progressive neuromuscular condition designated by a long-term muscle deterioration significantly affecting pulmonary and cardiac function and leading to premature death. However, whether TRIM32 plays a role in muscle wasting in sepsis needs to be shown.

Beyond that, a study by Volodin et al. showed that the paired box 4 (PAX4) transcription factor regulates the expression of enzymes that are required for myofibril degradation in later phases of denervation-induced atrophy [136]. All PAX family transcription factors play crucial roles in embryonic development and organogenesis. PAX4 in particular is essential for the differentiation of pancreatic β cells in mice, and mutations can lead to type 2 diabetes in humans [172,173,174]. PAX4 increases the expression of the p97/VCP ATPase complex in muscle [136]. p97/VCP extracts proteins from immobile cellular structures for proteasomal degradation [175] and catalyzes myofibril disassembly by release of ubiquitinated proteins to the cytosol [176]. The disassembled structure of myofibrils may in turn enhance the substrate availability for E3 ubiquitin ligases such as TRIM32, accelerating the degradation of thin filaments (Figure 2). Accordingly, downregulation of PAX4 is paralleled by a decreased expression of p97/VCP and a reduced myofibrillar breakdown in denervated mouse muscles [136].

### 3.4. Neural Precursor Cell Expressed Developmentally Down-Regulated Protein 4 (NEDD4)

The role of the ubiquitously expressed E3 ubiquitin ligase neural precursor cell expressed developmentally down-regulated 4 (NEDD4) in skeletal muscle atrophy is not well defined. NEDD4 is a HECT (Homologous to the E6-AP Carboxyl Terminus) domain ubiquitin ligase that is increased in skeletal muscles after denervation and unloading [177]. Increased NEDD4 expression was described in muscles in response to hindlimb unloading [177,178,179] and long-term denervation [180], but not in fasting or diabetes [177]. The activity of NEDD4 is regulated by posttranslational modifications including tyrosine phosphorylation by SRC [181], which activates NEDD4 activity, and by auto-ubiquitination, which causes NEDD4 oligomerization and inhibits its activity [182]. NEDD4 is localized to the sarcolemmal region of muscle fibers. Its first discovered target, Notch1, mediates satellite cell proliferation after muscle injury [177]. NEDD4 expression is also induced by PAX4 [136]. *Nedd4* knockout mice showed a reduction of IGF-1 and insulin signaling, delayed embryonic development, reduced growth and body weight as well as neonatal lethality [183]. Skeletal muscle-specific *Nedd4* knockout mice were protected against denervation-induced muscle atrophy [184]. Whether NEDD4 plays a role in muscle atrophy in sepsis or ICUAW is not known.

### 3.5. TNF Receptor-Associated Factor 6 (TRAF6)

TNF receptor-associated factor 6 (TRAF6) is a member of the TRAF family of adaptor proteins, possesses E3 ubiquitin ligase activity and predominantly promotes lysine 63 (K63)-linked polyubiquitin chains [185]. TRAF6 is upregulated in skeletal muscle in response to denervation, starvation and cancer cachexia. Skeletal muscle-specific deletion of *Traf6* protects against denervation-induced muscle atrophy in mice. This is attributable to a downregulation of atrogin-1/MAFbx and MuRF1 as well as a reduction of NF-κB, JNK, p38 MAPK and AMPK signaling pathways in the *Traf6* knockout mice. Besides that, TRAF6 inhibition was shown to inhibit cancer cachexia [186].

### 3.6. Muscle Ubiquitin Ligase of the SCF Complex in Atrophy-1 (MUSA1) and Others

Other E3 ubiquitin ligases have also been shown to be involved in skeletal muscle protein breakdown. Milan et al. found that a group of E3 ubiquitin ligases were upregulated in skeletal muscle of denervated or fasted mice, and were blunted in mice with a skeletal muscle-specific deletion of *FoxO1*, *-3* and *-4* [187]. These E3 ubiquitin ligases include MUSA1 (muscle ubiquitin ligase of the SCF complex in atrophy-1), Fbxo31 and SMART (Specific of Muscle Atrophy and Regulated by Transcription, Fbxo21). In addition, other factors of the ubiquitin conjugation/deconjugation machinery, proteasome subunits as well as ALP-related genes, were reduced in theses triple knockout mice [187]. These data suggest a complex regulation of protein degradation and protein synthesis by FoxO1, -3 and -4, and therefore by IGF-1, which mediates its effects through these transcription factors. Because the individual FoxO-transcription factors have specific targets, this regulation is even more complex. For example, FoxO3 overexpression was shown to induce MUSA1 but no other E3 ubiquitin ligase in myotubes. FoxO1 and FoxO3 were shown to bind to the promoter regions of MUSA1 and SMART, and deletion FoxO3 attenuated the induction of SMART, but not other E3 ubiquitin ligases. Because IGF-1 signaling is reduced by inflammatory cytokines and in sepsis, it is tempting to speculate that this mechanism contributes to muscle atrophy in sepsis. However, this hypothesis needs to be proven.

The role of DUBs in muscle wasting is less defined; however, due to the general function of DUBs, it can be assumed that DUB action may counteract muscle protein breakdown (reviewed in [188]). Further details on the regulation of activity and function of E3 ubiquitin ligases and related pathways in muscle atrophy in general have recently been reviewed elsewhere [189].

## 4. Role of Proinflammatory Cytokines in Inflammation-Induced Muscle Wasting

**Figure 3:**
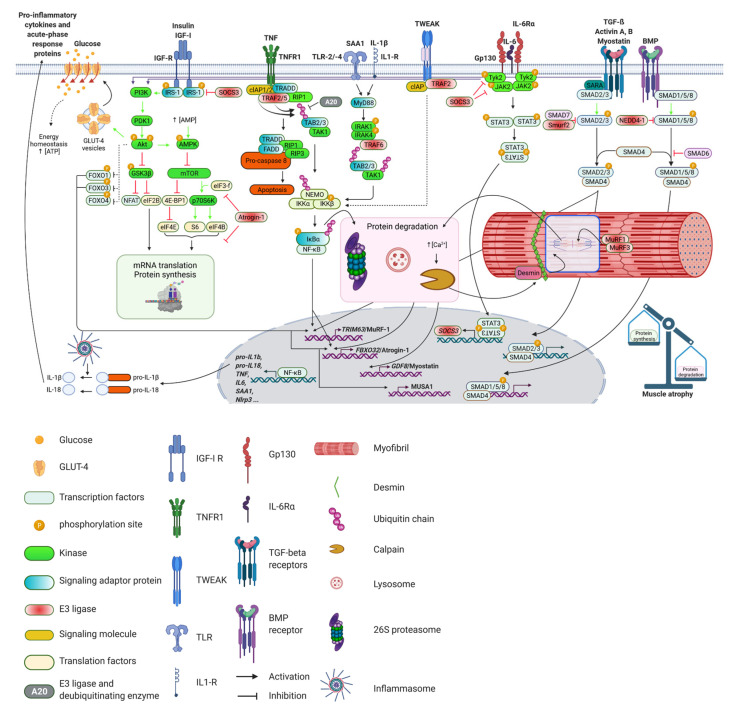
Signaling pathways involved in inflammation-induced skeletal muscle atrophy. BMP R: Bone Morphogenetic Protein (BMP) Receptor; Calp: Calpain; Fn14: Fibroblast Growth Factor-Inducible 14; GP130: Glycoprotein 130; GSK-3β: Glycogen Synthase Kinase 3β; IL-1β: Interleukin-1β; IL-1 R: Interleukin 1 Receptor; IL-6: Interleukin-6; IGF R: Insulin-like Growth Factor (IGF) I Receptor; P: Phosphorylation; MuRF: Muscle RING finger 1; NF-κB: nuclear factor ‘kappa-light-chain-enhancer’ of activated B-cells; SAA1: Serum Amyloid A1; STAT: Signal Transducer and Activator of Transcription; TGF-β R: Transforming Growth Factor β (TGF-β) Receptor; TLR: Toll-like Receptor; TNF R: Tumor Necrosis Factor (TNF) Receptor; TWEAK: Tumor Necrosis Factor-Related Weak Inducer of Apoptosis; SMAD: Small Mothers Against Decapentaplegic. For further details, please refer to the text. Created with https://biorender.com/.

Meta-analysis revealed that higher levels of circulating inflammatory markers are associated with a marked decrease in skeletal muscle strength and mass [190]. Among the best-studied proinflammatory cytokines promoting muscle atrophy are tumor necrosis factor (TNF), interleukin-6 (IL-6), interleukin-1 (IL-1), IFN-γ and TNF-like weak inducer of apoptosis (TWEAK). These cytokines are elevated in sepsis and may together cause muscle wasting, probably by increasing NF-κB or by causing the release of other cytokines [191,192]. In addition, skeletal muscle acts as a secretory immunogenic organ, producing and releasing numerous cytokines and other proteins referred to as myokines. Myokines mediate cross talk to several organs, such as the brain, adipose tissue and liver, and may influence their function [193]. IL-6 is the founding member of the myokine class of proteins. However, in systemic inflammation, skeletal muscle not only synthesizes and releases IL-6 [194] but also other cytokines, such as IL-1β [195,196], and acute phase proteins, such as serum amyloid A1 (SAA1) [43,194]. Interestingly, the levels of circulating cytokines are not necessarily associated with their tissue levels. The significance of this observation and the question if differences exist between systemic and local effects of cytokines warrants further investigation. In the following paragraph, the role of key inflammatory cytokines and their signaling pathways in inflammation-induced muscle wasting will be described. The main pathways are illustrated in Figure 3.

### 4.1. Tumor Necrosis Factor (TNF)

TNF, initially named cachectin [197], promotes skeletal muscle wasting in various pathological conditions, such as immobilization [138] and cachexia [198,199]. Early studies showed that TNF treatment of cultured myotubes caused a reduction in total protein content and a decrease in the myotube diameter indicative of atrophy [200]. Rats treated with recombinant TNF or IL-1β showed a significant reduction in total muscle protein content and a reduced expression of myofibrillar proteins [201,202]. ICU patients displayed increased TNF serum levels [203], and maximal TNF plasma levels were found to be higher in patients who developed ICUAW as compared with control subjects [204]. Importantly, the administration of an anti-TNF antibody inhibited the sepsis-induced increase in muscle proteolysis in rats, indicating that TNF is an important regulator of muscle protein breakdown during sepsis [205]. The atrophic effects of TNF are closely related to NF-κB and its downstream targets atrogin-1/MAFbx and MuRF1. TNF increases the expression of atrogin-1/MAFbx and MuRF1 in mouse skeletal muscle in vivo and in C2C12 myotubes in vitro, which is accompanied by myotube atrophy [206]. Likewise, TNF-induced atrophy was shown to be accompanied by an increase in atrogin-1/MAFbx and decrease the translation initiation factor eIF3f abundance in rat L6 and mouse C2C12 myotubes. These data suggest that TNF not only increases proteolysis but also decreases protein synthesis in myocytes [207].

TNF binds to the sarcolemmal TNF receptor 1 (TNFR1) or TNFR2, which in turn activates the NF-κB and PI3K/Akt signaling pathways as well as ubiquitin mediated proteolysis [208]. TNFR1 was shown to stimulate apoptosis via interaction with the TRADD (tumor necrosis factor receptor type 1-associated DEATH domain protein), TRAF2 (TNF receptor associated factor 2) and RIP1 (receptor interacting serine/threonine kinase 1) complex [209]. Activation of this complex further leads to the activation of FADD protein (Fas associated via death domain) resulting in apoptosis. The activated TRADD complex also activates JNK (c-Jun N-terminal kinase) and the transcription factor AP-1. The activated complex promotes phosphorylation and subsequent proteasomal degradation of the IκBα protein, which leads to an activation of NF-κB (for further details on this pathway, please see below). Moreover, TNFR2 also activates NF-κB by stimulating the PI3K/Akt signaling pathway [210]. Importantly, the IGF1/Akt pathway controls myofibril growth and maintenance via the GSK-3β/nebulin/N-WASP pathway. GSK-3β-mediated inhibition of eukaryotic translation initiation factor 2b (eIF2B) leads to decreased protein synthesis. In general, Akt inactivates GSK-3β, which releases inhibition of eIF2B and promotes protein synthesis. GSK-3β was found to be required for skeletal muscle atrophy and muscle wasting [211]. GSK-3β also suppresses NFAT activity (Nuclear factor of activated T-cells), which inhibits myogenic differentiation [212]. In addition, it results in decreased formation of actin filaments by inhibition of nebulin and neuronal Wiskott–Aldrich syndrome protein (N-WASP) [211]. In summary, these data show that TNF perturbs protein homeostasis at different levels involving an increase in proteasome-mediated protein degradation and decreased protein synthesis.

### 4.2. Interleukin 6

IL-6 has pleiotropic functions in different tissues and regulates protein homeostasis in the skeletal muscle [212,213]. While an acute increase in systemic IL-6 promotes muscle growth and hypertrophy, its sustained elevation, as occurring in catabolic conditions such as cancer or sepsis, causes muscle atrophy [214]. However, the role of IL-6 signaling in sepsis-induced muscle atrophy is not well understood. IL-6 production is stimulated by other cytokines, such as TNF, and IL-1β. IL-6 contributes to systemic inflammation in critical illness and sepsis [215], and it increases protein breakdown and skeletal muscle atrophy [216]. It was also reported to increase the activities of the 26S proteasome as well as cathepsin B and L, which decreased the half-life of proteins in murine myotubes [217]. These data indicate that chronic elevation of circulating IL-6 is sufficient to cause muscle wasting. In addition, intra-peritoneal IL-6 injections were found to cause muscle atrophy in rats possibly due to an increased proteolysis [216]. Local IL-6 infusion into the tibialis anterior muscle was associated with a reduction in the total and myofibrillar protein content in rats [218]. These data suggest that IL-6 directly acts on skeletal muscle to cause atrophy. In support of this, IL-6 treated C2C12 myotubes showed a lower mTOR and 4EBP-1 phosphorylation and an increased STAT3 phosphorylation and atrogin-1/MAFbx expression, which was paralleled by a reduced myotube diameter. This indicates that IL-6 increases atrogene gene expression and diminishes mTOR signaling and thereby protein synthesis in muscle [219]. However, other authors show only little effects of IL-6 on skeletal muscle both in vivo and in vitro [220], which may be explained by differences in IL-6 levels, shorter treatment durations or the pleiotropic nature of IL-6 in skeletal muscle. These data suggest that IL-6 increases protein degradation in muscle by activation of the proteasome and the lysosomal (cathepsin) pathway and decreases protein synthesis to cause atrophy.

IL-6 signaling is mediated either through binding to a membrane bound IL-6 receptor that facilitates homodimerization with the signal transducing receptor glycoprotein 130 (gp130, encoded by *Il6st*) [221] or through binding to a soluble IL-6 receptor, which then associates with gp130 [222]. Because other IL-6 cytokine family members such as IL-11, ciliary neurotrophic factor, leukemia inhibitory factor (LIF), oncostatin M, cardiotrophin 1, cardiotrophin-like cytokine and IL-27 also signal through gp130, they might as well play a role in sepsis-induced muscle wasting, but this needs to be proven [223]. Nevertheless, LIF has been reported to activate the JAK2/STAT3 signaling pathway to mediate skeletal muscle atrophy in a mouse model of cancer cachexia [224]. The activated IL-6R-gp130 complex binds and activates the Janus kinase (JAK) family of tyrosine kinases, primarily JAK1, JAK2 and tyrosine kinase 2 (TYK2), which phosphorylate the cytoplasmic tail of gp130, enabling the association of the STAT proteins, mainly STAT1 and STAT3. Phosphorylated STAT proteins dimerize and translocate to the nucleus where they control the expression of STAT target genes, such as Suppressor of Cytokine Signaling 3 (*SOCS3*) [225,226]. SOCS3 in turn acts as a negative feedback inhibitor of cytokine signaling by inhibition of JAK1, JAK2 and TYK2 [227]. SOCS3 also inhibits the Insulin/PI3K/Akt pathway as a substrate recognition component of an ubiquitin-protein ligase complex, degrading insulin receptor substrate 1 (IRS-1), which is essential for insulin signaling [228]. Insulin increases protein synthesis and decreases atrogene expression and protein degradation via the PI3K/Akt pathway [229,230]. This promotes muscle growth, and, inversely, perturbations of insulin signaling can aggravate muscle atrophy as frequently seen in critically ill patients [42]. Activated STAT3 also activates C/EBPδ (CCAAT-enhancer-binding protein d) causing an increased expression of myostatin (for further details, please see below), which consequently activates atrogin-1/MAFbx and MuRF1, and increases protein degradation [231]. Based on these data and together with the observation that increased IL-6 plasma level are a risk factor for Critical Illness Myopathy (CIM) correlated with peripheral nerve dysfunction [232], it is feasible that the IL-6/gp130/JAK/STAT pathway plays a role in sepsis-induced muscle atrophy. This, however, needs to be proven.

In this context, it is interesting to note that mouse models for denervation-induced muscle atrophy revealed the induction of standard- and immunoproteasomes indicating induction of inflammatory signaling. Here, immunoproteasome-deficient mice exhibited an altered activity and content of the remaining catalytic subunits, but the muscle loss was not attenuated [91].

### 4.3. Interleukin 1β

The interleukin (IL)-1 family comprises prototypic proinflammatory cytokines involved in the immune response to infection and injury, which consists of 11 family members. Interleukin-1α (IL-1α) and IL-1β are the best described and investigated cytokines of the IL-1 family. One of the most activated cytokines in sepsis is IL-1β [233,234,235]. Evidence for its role in muscle atrophy comes from in vitro and in vivo studies. For example, IL-1β treatment caused atrophy of differentiated C2C12 myotubes in a concentration-dependent manner, in vitro [195]. Treatment of rats with recombinant IL-1α resulted in an increased breakdown of myofibrillar proteins [236]. Chronic IL-1 infusion was reported to reduce muscle weight, which was associated with a decrease in protein content and protein synthesis in rat gastrocnemius muscle [237]. By contrast, when a IL-1 receptor antagonist was administered to septic or with endotoxin treated rats, muscle breakdown was attenuated [238]. Administration of an IL-1 antagonist also preserved muscle mass in a chronic abdominal sepsis rat model [239].

The expression, cleavage and secretion of IL-1β is tightly controlled. Inflammatory cytokines increase the expression of pro-IL-1β, which is the inactive proform of IL-1β. The conversion of pro-IL-1β to IL-1β and IL-1β secretion are mediated by caspase-1 activating inflammasomes [233,240]. Inflammasomes are multi-protein complexes of the innate immune system that have been shown to be involved in the pathogenesis of sepsis [241,242]. The nucleotide-binding domain (NOD)-like receptor (NLR) family are central components of the inflammasome. One of the best characterized NLR is NLRP3 [243,244]. The NLRP3 inflammasome comprises the sensor molecule NLRP3, the adaptor protein ASC and pro-caspase-1. In general, activation of the NLRP3 inflammasome requires two steps: priming and activation. The priming step occurs in response to inflammatory stimuli such as TLR4 agonists, which increases the expression of NLRP3 and pro-IL-1β via NF-κB. The activation step is mediated by pathogen-associated molecular patterns (PAMP) and damage-associated molecular pattern (DAMP). Upon activation, the NLRP3 protein interacts with ASC, which recruits pro-caspase-1 to form the NLRP3–ASC–pro-caspase-1 complex [245]. Proximity-induced autocatalytic activation of pro-caspase-1 to caspase-1 occurs upon its recruitment to this complex. In turn, active caspase-1 cleaves the cytokines pro-interleukin-1β (pro-IL-1β) and pro-IL-18 into their mature and biologically active forms [244]. NLRP3 is contained in muscle, and its activity is increased in myopathies [246]. NLRP3 knockout mice are incapable of activating IL-1β and therefore show reduced IL-1β serum levels at baseline as well as in sepsis. In accordance with the observation that IL-1β causes myocyte atrophy, *Nlrp3*-deficient mice are protected against sepsis-induced muscle atrophy. This was accompanied by an increased survival of septic *Nlrp3* knockout mice when compared with septic wildtype littermate controls [195]. IL-1β signal transduction is mediated by the IL-1 receptor, which is associated with IL-1 receptor associated kinase1 (IRAK1) that activates NF-κB [247]. This in turn increases atrogin-1/MAFbx and MuRF1 expression in myocytes [248,249]. Accordingly, septic *Nlrp3*-deficient mice showed a reduced activation of NF-κB, which was accompanied by a decrease in atrogin-1/MAFbx and MuRF1 expression when compared with wildtype littermate controls. Because *Nlrp3* deletion reduces mortality and sepsis-induced end-organ damage, such as muscle failure [195], cardiomyopathy [250,251] and acute lung injury, pharmacological NLRP3 inhibition might be beneficial in sepsis. Indeed, positive effects were reported for the NLRP3-inhibitors hemin, which protected against cecal ligation and puncture (CLP)-induced acute lung injury in mice [252], and scutellarin as well as glyburide, which improved survival of mice with bacterial sepsis [253,254]. Whether such an approach is applicable to critically ill human patients awaits further investigation. In addition, a reanalysis of a phase III randomized controlled trial that investigated the role of the IL-1 antagonist anakinra showed significant survival benefits in patients with sepsis [255]. However, whether this was related to a preservation in muscle mass and function is unknown.

### 4.4. Serum Amyloid A1 (SAA1)

The acute-phase response protein serum amyloid A1 (SAA1) is associated with inflammation during sepsis [43]. While the main source of SAA1 is the liver [256], inflammatory cytokines such as IL-6 increase SAA1 expression and secretion also in muscle [43,257,258]. SAA1 expression significantly increases and accumulates in the sarcolemma and interstitium of muscle from ICUAW patients, which was not observed in control subjects [43]. However, the role of SAA1 in ICUAW is not well defined [259]. Various tumors produce SAA1 [260,261], and it is associated with muscle wasting in cancer cachexia in mice [262]. SAA1 and IL-6 cooperate with each other to mediate angiotensin II-induced muscle atrophy, which is important for muscle wasting in heart failure patients [263]. In immune cells, SAA1 binds to several different receptors such as the purinergic receptor P2X7 (P2RX7), the scavenger receptor class b member 1 (SCARB1), the CD36 receptor (CD36), the VCP interacting membrane seleno-protein (VIMP) and Toll-like receptors (TLR) 2 and 4 [264,265,266]. SAA1 activates the NLRP3 inflammasome via P2RX7 causing an activation and release of IL-1β from mouse and human macrophages [264]. When SAA1 binds to the CD36 receptor, it increases *IL6* and *TNF* expression in rat macrophages and human embryonic kidney cells [265]. SAA1 was also shown to increase the pro-inflammatory cytokines IL-23α and TNF via TLR2, and to increase nitric oxide via TLR4 in mouse macrophages [267]. Recently, Hahn et al. identified the receptors and signaling pathway that mediate SAA1-induced muscle atrophy. They showed that SAA1 via the TLR2/TLR4/NF-κB signaling pathway mediates C2C12 myotube atrophy in vitro and sepsis-induced muscle atrophy in mice in vivo. They reported that SAA1 participates in a positive feedback loop of self-perpetuating inflammation in muscle during sepsis. Importantly, they reported that the IκB kinase-2 (IKK-2) inhibitor BMS-345541 that causes an inhibition of NF-κB reduces atrogin-1/MAFbx and MuRF1 expression, diminishes skeletal muscle atrophy and increases survival in septic mice [194]. It is therefore likely that SAA1 via activation of the TLR2/TLR4/NF-κB atrophy pathway is mechanistically important for ICUAW. However, the improved survival of BMS-345541 treated septic mice is possibly related to a systemic NF-κB inhibition. Further, experiments with myocyte-specific deletions of TLR2 and or TLR4 are needed to state that these receptors mediate sepsis-induced muscle atrophy in vivo. Whether SAA1 affects the proteasome function in muscle during sepsis is unknown.

### 4.5. TNF-Related Weak Inducer of Apoptosis (TWEAK)

The proinflammatory cytokine TNF-related weak inducer of apoptosis (TWEAK) also mediates skeletal muscle wasting. TWEAK binds to the cell surface receptor Fn14 (Fibroblast growth factor inducible 14), which activates TAK1 (Transforming growth factor beta activated kinase-1) and Akt. This results in phosphorylation and activation of IKKβ followed by NF-κB activation. Activated TAK1 also stimulates JNK-1 and p38 MAPK, which in turn activates AP-1. TWEAK increases the degradation of muscle proteins and leads to a reduction in the diameter of C2C12 cells and primary myotubes [268]. Similarly, chronic administration of TWEAK in vivo or muscle-specific overexpression of TWEAK in mice resulted in a loss of skeletal muscle mass. The TWEAK-mediated loss of muscle mass is mediated by several mechanisms, such as an activation of NF-κB or the UPS as well as an inhibition of the Akt pathway. For example, TWEAK caused a rapid and sustained increase in both DNA binding and transcriptional activity of NF-κB in myotubes and activated NF-κB in skeletal muscle of mice in vivo. In addition, treatment of myotubes with TWEAK reduced the phosphorylation of Akt, which was paralleled by a reduced phosphorylation of GSK-3β, mTOR, p70S6K and FOXO1. These data suggest that TWEAK inhibits the activity of the PI3K/Akt pathway [269]. Finally, TWEAK-induced MyHC-degradation and myotube atrophy was attenuated by inhibition of MuRF1, autophagy or caspase-3 [270], suggesting that these factors are involved in TWEAK-mediated muscle atrophy. In summary, these data suggest that TWEAK mediates muscle atrophy by an increased protein degradation and a reduced protein synthesis, resulting in a perturbed protein homeostasis and muscle wasting. The role of TWEAK in ICUAW is uncertain.

### 4.6. Transforming Growth Factor (TGFs) and Its Signaling Pathways

The precise function of the transforming growth factor (TGF) cytokine family in inflammation-induced muscle wasting is not well understood. However, evidence suggests a role of TGF family in sepsis-induced muscle wasting, i.e., TGF-β1 serum levels are increased in sepsis [271,272]. Because TGF-β1 inhibits the release of proinflammatory cytokines, such as IL-1 and TNF from monocytes and macrophages, it may be important for immunosuppression often seen in sepsis [273,274]. TGF-β1 also increases the production of immunosuppressive factors, such as the soluble TNF receptors (sTNFRs) and the IL-1 receptor antagonist (IL-1Ra) [275]. In addition, TGF-β1 is involved in muscle atrophy. For example, overexpression of TGF-β1 in muscle causes skeletal muscle atrophy in mice [276]. TGF-β1 preferentially mediates atrophy of fast twitch/type II myofibers [277]. Moreover, myostatin, which is a TGF-β family member, induces atrophy of fast twitch/type II myofibers [278]. Because type II myofibers are mainly affected in ICUAW, TGF-β1 might be involved in this pathology. By contrast, clinical studies showed that TGF-β1 inhibitors reduce muscle atrophy. For example, a TGF-β1 neutralizing antibody attenuated muscle atrophy and improved muscle regeneration [279]. The TGF-β receptor I (TβRI) antagonist Ki26894 increased muscle weight and strength in wildtype mice and diminished muscle atrophy in a transgenic mouse model of muscle dystrophy [280]. A role of TGF-β1 in skeletal muscle atrophy is further supported by the observation that increased TGF-β1 expression is associated with muscle atrophy in muscular dystrophy patients [281]. Together, these data suggest that TGF-β1 may be involved in inflammation-induced skeletal muscle atrophy.

The TGF family is mainly divided into two subfamilies with opposing effects on muscle mass. In general, TGF-β/myostatin/activin are seen as negative regulators, and BMPs (Bone Morphogenic Proteins)/GDF (Growth and Differentiation Factors) are judged as positive regulators of muscle mass [282]. TGF-β binds to the TβRI and TβRII complex to activate both canonical SMAD-dependent and non-canonical signaling pathways, such as PI3K/Akt, MAPK pathways (ERK, JNK and p38 MAPK) that activate protein synthesis and muscle growth [283,284]. TGF-β/myostatin/activin mainly activates SMAD2 and SMAD3 that are pro-catabolic. By contrast, BMP ligands predominantly recruit the SMAD1, SMAD5 and SMAD8 that elicit an anabolic transcriptional program. SMAD4 is shared by both pro-anabolic and pro-catabolic SMADs and can be a limiting factor for SMADs’ downstream effects. In terms of muscle mass control, Myostatin/GDF8 is the best described TGF-family member [285], next to GDF11 and Activin A [286,287]. Myostatin is a myokine, and its autocrine and paracrine effects limit skeletal muscle growth. It is involved in muscle mass regulation during embryonic development and plays a key role in (patho-)physiologic adaptations of skeletal muscle mass. Myostatin is highly conserved across species, and loss of myostatin function leads to skeletal muscle hypertrophy, hyperplasia and eventually a massive increase in muscle mass in humans, mice and cattle [288]. By contrast, myostatin overexpression has been shown to be involved in muscle atrophy during different diseases such as chronic heart failure or chronic obstructive pulmonary disease [289]. Upon myostatin binding, atrogin-1/MAFbx and genes involved in the degradation of several anabolic factors (ribosomal proteins, translation initiation factors, MyoD, desmin and vimentin) are upregulated [290] and the Akt/mTORC1 pathway is inhibited [291]. Furthermore, myostatin has been found to be associated with cancer cachexia, and its expression is stimulated through the JAK/STAT pathway [192]. Binding of myostatin to ActRIIB results in the phosphorylation of SMAD2/3 and activation of SMAD signaling [292], which reduces levels of Akt phosphorylation [287], consequently activating caspase-3 and FoxO and resulting in increased protein degradation [192]. Myostatin administration is sufficient to induce cachexia in mice through ActRIIB signaling [293]. However, whether or not myostatin is involved in inflammation-induced muscle atrophy has not been investigated so far. The activity of the TGF-β-, BMP- and activin A-signaling pathways is also regulated by the HECT E3 ubiquitin ligase SMURF1 (Smad ubiquitination regulatory factor 1) via interaction and proteasomal degradation of the signal transducing SMAD1, SMAD5 and SMAD4 proteins in vitro [294]. SMURF1 also indirectly interacts with TβRI via SMAD7 and thereby facilitates the degradation of TβRI [295]. As a key downstream effector of activin-mediated ActRII signaling, SMURF1 increases proteasome-dependent degradation of sarcoplasmic reticulum calcium ATPase (SERCA2a), which is a critical determinant of cardiomyocyte function [296]. Because the TGF-β family significantly affects muscle mass and function, it can be assumed that modulation of TGF-β signaling by SMURF1 also affects muscle atrophy in sepsis. However, this has not been proven so far.

TGF-β via the synergistic action of FOXO3a and SMAD3 increases the expression of MuRF1/*Trim63* in vitro [297]. Similarly, Activin A negatively regulates muscle mass [298]. Interestingly, the non-canonical TGF-β pathway involving TAK1-p38 MAP kinase can also be activated by Activin A treatment in cells and in vivo, leading to atrogin-1/MAFbx-mediated myotube atrophy [299]. Recent reports showed that the canonical NF-κB and angiotensin II pathways mediate the TGF-β effects in cells and in vivo [300]. Conversely, the BMP pathway regulates hypertrophy by repressing the E3 ubiquitin ligases atrogin-1/*Fbxo32*, MuRF1/*Trim63*, MUSA1/*Fbxo30* and through activation of mTORC1 consequently protein synthesis [301,302,303]. Altogether, these data implicate that the net balance between the pro-anabolic and pro-catabolic TGF-β/BMP pathways are important for determination of skeletal muscle mass. However, the multitude of ligands and receptors as well as the downstream canonical and non-canonical signaling pathways warrant further analyses to state their individual contribution in sepsis-induced muscle wasting.

### 4.7. Signaling Pathways That Mediate Inflammation-Induced Muscle Wasting

#### 4.7.1. Nuclear Factor-κB (NF-κB)-Signaling

The transcription factor NF-κB (nuclear factor ‘kappa-light-chain-enhancer’ of activated B-cells) is a central component in septic shock. It is a key regulator of inflammatory responses, and its activation is an important step in inflammation-induced muscle wasting [304,305,306]. NF-κB represents a family of inducible transcription factors, which regulates a large array of genes involved in different processes of the immune and inflammatory responses [307]. Inhibition of NF-κB alleviates cytokine-induced muscle atrophy, indicating that NF-κB is directly involved in inflammatory muscle wasting [308]. As stated above, the properties of the inflammatory cytokines TNF, IL-1β and IL-6 and acute phase response proteins such as SAA1, which cause muscle wasting, have mainly been attributed to receptor-mediated activation of NF-κB. In turn, NF-κB increases the expression of several proinflammatory cytokines, which initiates a self-augmenting positive feedback loop, and enhances protein degradation [309].

The NF-κB family is composed of five structurally related members, including NF-κB1 (also named p50), NF-κB2 (also named p52), RelA (also named p65), RelB and c-Rel. Transcriptionally active NF-κB dimers are formed by combinatorial association of these five subunits. The NF-κB subunits p50 and p52 are generated by proteasomal cleavage of the p105 and p100 precursor proteins, respectively [310,311,312]. Although the proteasome is involved in cleavage of both p105 and p100, the processing of p105 is primarily constitutive, whereas the processing of p100 is primarily inducible [313]. The NF-κB family mediates transcription of target genes by binding to a specific DNA element, κB enhancer, as various hetero- or homo-dimers [314]. Whether there is a specific role for the different NF-κB family members or particular hetero- or homo-dimers in ICUAW is unknown. In the majority of cells, NF-κB exists in an inactive form in the cytoplasm bound to the inhibitory protein IκB with IκBα being the best-studied IκB family member [315]. In response to inflammatory cytokines (e.g., TNF, IL-1β, IL-6), IκB kinase is activated and phosphorylates IκB, which leads to its ubiquitination and degradation by the proteasome [316]. Especially, K48- and K63-linked ubiquitin chains play a role on several levels in NF-κB activation [317]. Importantly, transgenic mice expressing a mutant isoform of ubiquitin that interferes with ubiquitin chain assembly in a dominant negative manner (K48R mutant ubiquitin) displayed a disrupted NF-κB activation and improved survival in response to lipopolysaccharide (LPS)-induced toxic shock [318]. Once activated, NF-κB translocates to the nucleus and stimulates the expression of its target genes, especially atrogin-1/MAFbx and MuRF1, which in turn mediate muscle atrophy [194,319]. Therefore, many approaches were undertaken to reduce the NF-κB activity in various disease models, e.g., miss-expression of IκB in muscle that inhibits NF-κB attenuated denervation-induced atrophy in mice [304]. The absence of IκB kinase-β also reduced atrophy in response to denervation in mice [305]. Synthetic double-stranded oligodeoxynucleotides, which block NF-κB binding to its promoter elements, have been shown to inhibit tumor cachexia in a mouse model [320]. Activation of NF-κB, through muscle-specific transgenic expression of activated IκB kinase beta (MIKK), causes profound muscle wasting mainly due to increased UPS-mediated protein degradation. Indeed, MIKK mice showed an increased expression of MuRF1. By contrast, no overt phenotype was seen upon muscle-specific inhibition of NF-κB through expression of IκBα super-repressor (MISR). However, denervation- and tumor-induced muscle loss were substantially reduced and survival rates improved by NF-κB inhibition in MISR mice [304]. Importantly, Hahn et al. reported that the IκB kinase-2 (IKK-2) inhibitor BMS-345541 that in turn inhibits NF-κB reduces atrogin-1/MAFbx and MuRF1 expression, diminishes skeletal muscle atrophy and increases survival in septic mice [194]. Finally, because proteasome inhibitors suppress IκB degradation, they interfere with the NF-κB pathway, this in turn prevents NF-κB activation [321]. Since atrogin-1/MAFbx and MuRF1 are NF-κB target genes, proteasome inhibition is expected to prevent muscle atrophy by maintaining NF-κB in an inactive state and thus preventing upregulation of atrogin-1/MAFbx and MuRF1. Indeed, the proteasome inhibitor MG132 was shown to attenuate immobilization-induced atrophy. Likewise, the proteasome inhibitor bortezomib has been shown to reduce atrophy in response to denervation [322]. For more information about the effects of proteasome inhibitors on muscle atrophy, please see below.

#### 4.7.2. JAK/STAT-Signaling

The role of the JAK/STAT signaling pathway in muscle pathophysiology has recently been reviewed in detail and was described with the IL-6 pathway [323].

The JAK/STAT pathway is activated by type I (IFN-α/β), type II (IFN-γ), IL-2, and IL-6 receptor stimulation [324]. IL-6 binding to the IL-6r-gp130 receptor complex results in the recruitment to the intracellular domain of the receptor, and subsequent activation of the JAK tyrosine kinase. After binding, JAK proteins undergo a conformational change, dimerize and activate the STAT proteins through phosphorylation. Subsequently homo- or hetero-dimerization of STAT proteins is followed by translocation to the nucleus [323]. STAT transcriptional activation contributes to muscle wasting through various mechanisms. It stimulates C/EBPδ expression and activity, which in turn increases myostatin, atrogin-1/MAFbx1, MuRF1 and caspase-3 expression in myofibers, therefore enhancing muscle proteolysis [192,218,325]. Moreover, increased myostatin expression resulting from STAT/C/EBPδ activation suppresses postnatal myogenesis, which in turn may negatively affect muscle mass maintenance [192]. Furthermore, STAT3 was documented to regulate gene transcription by interaction with FoxO and NF-κB [326,327].

#### 4.7.3. MAPK Signaling

The MAPK pathway controls growth and stress responses in a wide variety of different cell types, including skeletal muscle. MAPK signaling is activated by cellular stress, growth factors, and pro-inflammatory cytokines (e.g., IL-1 and TNF) [328]. The MAPK family of proteins consists of four distinct signaling pathways, namely, extracellular signal-regulated kinases 1 and 2 (ERK1/2), p38 MAPK, c-Jun NH2-terminal kinases (JNK), and ERK5 [329]. p38 MAPK mediates upregulation of atrogin-1/MAFbx and MuRF1 in response to TNF by an unknown mechanism [206]. IL-1 signaling has also been shown to stimulate phosphorylation of p38 MAPK, leading to increased atrogin-1/MAFbx expression, independent of Akt/FoxO signaling [248]. Furthermore, p38 phosphorylates MRF4 (myogenic regulatory factor 4), thus inhibiting the expression of selective myogenic genes in late myogenesis, and antagonizes the JNK proliferation-promoting pathway [330]. JNK mediates AP-1 activation, which has been implicated in muscle atrophy responses [331]. TNF treatment caused an increase in p-ERK in differentiating myoblasts, which was accompanied with reduced MyoD and MyoG levels, as well as reduced MyHC protein content. Administration of the ERK inhibitor PD98059 to C2C12 cells prevented this inhibitory effect of TNF on myogenic differentiation [332].

#### 4.7.4. TFEB/TFE3-Signaling

As already mentioned, *TRIM63*/MuRF1 mRNA and protein expression are rapidly and strongly increased in various physiological and pathological atrophy conditions [137]. The strong transcriptional regulation during atrophy together with the multitude of its target proteins highlights the importance of MuRF1 in muscle homeostasis. The expression of MuRF1/*TRIM63* is regulated by several signaling pathways converging onto multiple transcription factors, such as the FoxO protein family [333,334], myogenin, and NF-κB [335]. Recently, the basic helix-loop-helix leucine zipper (bHLH-LZ) transcription factor EB (TFEB) and its microphthalmia/transcription factor E (MiT/TFE) family member TFE3 were shown to regulate *Trim63*/MuRF1 expression in vitro [336,337]. Importantly, TFEB- and TFE3-induced *TRIM63* expression was shown to be tightly regulated by a transcriptional network comprised of the class IIa histone deacetylases (HDAC) 4, HDAC5 and HDAC7 and the protein kinase D-family members PKD1, PKD2 and PKD3. In this pathway, the class IIa HDACs physically interact with and inhibit the activity of TFEB and TFE3, whereas the PKDs associate with and phosphorylate these HDACs, resulting in their nuclear export and derepression of TFEB and TFE3 followed by an increase in *Trim63*/MuRF1 expression. This pathway may at least partially be involved in heart failure-induced muscle wasting (cardiac cachexia) [336]. Whether it plays a role in inflammation-induced muscle atrophy warrants further investigation.

#### 4.7.5. IGF/PI3K/AKT Signaling

The insulin-like growth factor-1(IGF-1)/phosphatidylinositol 3-kinase (PI3K)/Akt/mammalian target of rapamycin (mTOR) pathway is one of the best characterized pathways, which regulates protein turnover. Evidence suggests that the serine/threonine kinase Akt (also called Akt1 and PKB, for protein kinase B) increases skeletal muscle mass by activating anabolic and suppressing catabolic pathways. IGF-1 induces hypertrophy of differentiated myotubes in vitro [229] and skeletal muscle in vivo since transgenic mice that overexpress IGF-1 in the skeletal muscle display an increased muscle size [338,339]. IGF-1 via its receptor activates PI3K. PI3K phosphorylates the membrane phospholipid phosphatidylinositol 4,5-bisphosphate to produce phosphatidylinositol 3,4,5-trisphosphate [340], creating a lipid binding site on the cell membrane for Akt [341]. The subsequent translocation of Akt to the membrane facilitates its phosphorylation and activation by the kinase PDK-1 [342]. Direct and indirect targets downstream of Akt include mTOR, ribosomal S6 kinase (p70S6K) and PHAS-1 (4EBP-1), key regulatory proteins involved in translation and protein synthesis [229]. Akt-mediated phosphorylation of GSK-3β leads to its inhibition, which relieves the inhibitory effect of GSK-3β on the eukaryotic translation initiation factor 2B (eIF2B) [343]. Eukaryotic initiation factors (eIFs) and p70S6K regulate the translational capacity in skeletal muscle. The formation of the eIF4F complex is a rate-limiting step in initiation of mRNA translation [344], whereas phosphorylation of p70S6K promotes ribosomal biogenesis and translation [345]. Akt also phosphorylates and activates mTOR [346], which causes phosphorylation of p70S6K and PHAS-1/4E-BP1, activating protein synthesis and translation initiation, respectively [347]. In addition, Akt is known to prevent muscle loss by phosphorylating FoxO1 and FoxO3a, which prevents their translocation from the cytosol to the nucleus [333,348]. Recent studies have shown that FoxO transcription factors are necessary for both atrogin-1/MAFbx and MuRF1 gene expression [140,333]. Thus, activation of the PI3K/Akt signaling pathway in skeletal muscle inhibits the expression of atrogin-1/MAFbx and MuRF1 by inhibiting the activity of FoxO1 and FoxO3a transcription factors [333,349]. The activity of the PI3K/Akt/FoxO pathway has been shown to be suppressed by the deubiquitinating enzyme ubiquitin-specific protease 1 (USP1) in muscle. Specifically, USP1 removes K63-linked polyubiquitin chains on Akt, which reduces Akt phosphorylation at theonine 308 and suppresses PI3K/Akt signaling when cellular energy levels are low. USP1-mediated inhibition of the PI3K/Akt pathway may contribute to muscle wasting, but whether it plays a role in ICUAW needs to be investigated [350]. Proinflammatory cytokines such as TWEAK have been shown to decrease Akt activity, which ultimately reduces protein synthesis and increases atrogin-1/MAFbx and MuRF1 expression via FoxO transcription factors [269].

In addition, insulin via insulin receptor increases phosphorylation of IRS-1, which is the predominant signaling mediator of this pathway. IRS-1 in turn activates Akt, GSK-3β and Akt substrate 160 (AS160) to induce translocation of GLUT4, the most important glucose transporter in muscle cells, to the cell membrane facilitating glucose uptake and energy production. In critically ill septic patients, insulin resistance is frequently observed, leading to a perturbed PI3K/Akt/GLUT4 pathway with decreased availability of glucose and energy, a decreased protein synthesis and an increased protein degradation [42]. The precise mechanism of how proinflammatory cytokines mediate insulin resistance is not well understood. However, IL-6 has been shown to increase the expression of SOCS3, which in turn interacts with IRS-1 and mediates its proteasomal degradation [228]. This mechanism might in part explain insulin resistance in septic patients occurring at the post-receptor level.

### 4.8. Involvement of Myogenic Regeneration in Muscle Atrophy of Critically Ill Patients

Skeletal muscle has a remarkable regenerative capacity. However, long-term sequelae of ICUAW persist for up to 5 years after ICU or hospital discharge, which indicates a significant defect in myogenic regeneration [17,19]. Previously, a diminished regenerative capacity was shown in muscle of ICUAW patients and septic mice. This was accompanied by a reduced number and a defective activation of adult muscle stem cells called satellite cells as well as an impaired myogenic regeneration [351,352,353]. Satellite cells are the tissue-residing stem cells of skeletal muscle, providing myonuclei for postnatal muscle growth and for maintenance and regeneration in adults [354]. They are located between the basal lamina and the plasmalemma of myofibers [355]. Once activated, satellite cells proliferate, differentiate and fuse to multinucleated myofibers [354]. This well-regulated process of myogenesis requires the sequential expression of myogenic transcription factors [356], proteins involved in myoblast fusion [357,358] and contractile proteins [359]. The role of myogenesis in maintaining skeletal muscle size is well described [360], but its importance in critical illness is uncertain. Importantly, TGF-β superfamily members, such as TGF-β, myostatin and activin, were shown to inhibit myogenic differentiation in vitro and in vivo [361,362,363]. By contrast, studies using the TβRI specific inhibitor SB-431542 or overexpression of a dominant negative TβRII in myoblasts revealed that TGF-β receptors are essential for myogenesis [364,365]. Recently, an increased TGF-β signaling and elevated TβRII levels were reported in muscle of critically ill patients suggesting their involvement in muscle atrophy [366,367]. TGF-β binds to the TβRI and TβRII complex to activate both canonical SMAD-dependent and non-canonical signaling pathways, such as Akt, which activates protein synthesis and muscle growth [283]. Additionally, and important for this review, is a study by Kitajima et al., who investigated the function of the proteasome in satellite cells using mice lacking the crucial proteasomal 19S component, Rpt3. A satellite cell-specific ablation of Rpt3 resulted in a decreased proteasome activity. Proteasome dysfunction in Rpt3-deficient satellite cells impaired their ability to proliferate, survive and differentiate, resulting in defective muscle regeneration. Mechanistically, they found that the proteasome dysfunction caused an activation of the p53 pathway, which caused cell-cycle arrest [368]. However, whether or not muscle regeneration plays a role in sepsis-induced muscle wasting and whether satellite cells and their contribution to myogenic differentiation and regeneration are involved in this phenotype need further analyses.

### 4.9. Is the Ubiquitin Proteasome System a Suitable Target to Prevent Inflammation-Induced Muscle Wasting?

Given the role of UPS-dependent protein degradation in skeletal muscle atrophy [369], several groups tested if proteasome inhibitors are effective in this pathology [370,371,372,373]. For example, the proteasome inhibitors MG132 and *N*-acetylleucyl-leucyl-norleucinal (LLN) suppressed proteolysis in isolated rat skeletal muscles [370]. At baseline, these proteasome inhibitors inhibited protein breakdown by up to 50%, which was even more pronounced in atrophying muscles. Importantly, MG132 significantly reduced the increase in hind limb and diaphragm muscle proteolysis in CLP-operated septic rats [370,374]. Likewise, the proteasome inhibitors N-acetyl-L-leucinyl-L-leucinal-L-norleucinal (LLnL) and lactacystin also inhibited protein breakdown in muscles from septic rats in vitro [372], and systemic MG132 treatment decreased the inflammatory response and prolonged survival in CLP-induced sepsis in mice in vivo [375]. These studies support the hypothesis that the proteasome system is largely responsible for inflammation-induced muscle proteolysis. However, these studies did not measure muscle force generation or determine if proteasome inhibitors had any effect on preserving muscle strength in sepsis. Further analyses by Supinski et al. tested if the proteasome inhibitors MG132, epoxomicin and bortezomib prevent endotoxin-induced proteolysis in the diaphragm muscle of rats and if this has favorable effects on muscle strength. Although all of these proteasome inhibitors attenuated endotoxin-induced proteolysis in the diaphragm muscle, they did not prevent the reduction in diaphragm force production [376]. Therefore, these proteasome inhibitors are unlikely to preserve muscle function in toxic shock patients. However, when the proteasome inhibitor bortezomib was administered 1 hour before CLP-surgery, it not only reduced serum levels of inflammatory cytokines but also increased the survival of septic mice [377]. The proteasome inhibitors MG132 and lactacystin also decreased the inflammatory response and prolonged survival in CLP-operated mice [375,378]. This survival benefit is probably related to systemic anti-inflammatory effects as opposed to direct effects on muscle proteolysis. In this regard, proteasome inhibitors were found to be effective in chronic inflammatory diseases, such as rheumatoid arthritis and inflammatory bowel disease [379,380,381], and the anti-inflammatory effects are again thought to be related to an inhibition of NF-κB [311,382]. In general, bortezomib is well tolerated by patients, but it is also associated with toxicity, such as thrombocytopenia, nausea, diarrhea, fatigue, peripheral neuropathy and generalized weakness [383]. Prevention and treatment of muscle atrophy is often a long-term approach; therefore, side effects of any therapy are likely to occur. Because the proteasome mediates degradation of a multitude of proteins involved in critical biological processes, its inhibition may promote the formation of protein aggregates that may cause proteotoxic and adverse effects (see next chapter). Indeed, proteasome inhibition by MG132 was shown to cause an accumulation of ubiquitin-conjugated proteins in rat skeletal muscle [370]. Occurrence of protein aggregates have also been described in patients with inclusion body myopathy associated with Paget disease of the bone and frontotemporal dementia (IBMPFD), which is an autosomal dominant disorder attributed to mutations in p97/VCP [384]. Several missense mutations in p97/VCP have been identified in IBMPFD patients, and most of them caused accumulations of ERAD substrates [385]. These accumulations are associated with the IBMPFD phenotype that is characterized by adult-onset proximal and distal muscle weakness and premature frontotemporal dementia manifesting in muscle, brain and bone. Additionally, most studies used a preventive rather than a therapeutic approach to investigate effects of proteasome inhibitors, such as bortezomib, which is impossible to be translated into the clinical setting. Taken all these findings together, the application of pan-reactive proteasome inhibitors to treat ICUAW appears questionable. However, immunoproteasome-specific inhibitors that have been shown to be immunosuppressive in mouse models are in phase I trials and may be clinically approved for treatment of hematopoietic malignancies soon. Their possible benefit in muscle wasting has to be evaluated very carefully in the future [370,386].

Due to the ubiquitous distribution of the proteasome and its involvement in multiple cellular processes that are not restricted to striated muscles, its unselected inhibition in patients may cause more harm than good. One way around this would be to inhibit a specific UPS-related factor that is restricted to striated muscles and plays a central role in muscle atrophy. Because the role of the muscle-restricted E3 ubiquitin ligase MuRF1 in muscle atrophy is well described and many of its interaction partners are known, a MuRF1 inhibitor could be useful to prevent muscle atrophy. Indeed, the groups of S. Labeit and V. Adams performed an alpha-screen to search for small molecules that block the recognition of titin by MuRF1. This compound prevented dexamethasone-induced myotube atrophy in vitro and attenuated myofiber atrophy and contractile dysfunction in mouse muscle during cardiac cachexia in vivo by inhibition of UPS-dependent proteolysis and protection of de novo protein synthesis [387]. Further studies by the same groups showed that this MuRF1 inhibitor attenuated diaphragm dysfunction in chronic heart failure in mice [388], skeletal muscle atrophy and dysfunction in cancer cachexia in mice and skeletal muscle dysfunction in a rat model of heart failure with preserved ejection fraction [389]. Whether this inhibitor also prevents muscle wasting in sepsis is not known. Nevertheless, the inhibition of even tissue-restricted E3 ubiquitin ligases also needs to be assessed with caution. For example, the germ line deletion of *Trim54*/MuRF3 predisposed the heart for failure and rupture during acute myocardial infarction in mice [390]. In addition, it has been shown that a homozygous *TRIM63*/MuRF1 null mutation in combination with the heterozygous *TRIM54*/MuRF3 mutation leads to a myosin storage myopathy associated with skeletal muscle hypertrophy and hypertrophic cardiomyopathy in patients [391]. Likewise, the combined germ line deletion of *Trim63*/MuRF1 and *Trim54*/MuRF3 [151] as well as the deletion of *Trim55*/MuRF2 and *Trim54*/MuRF3 [153] caused a myosin storage myopathy of the heart and skeletal muscle in mice.

### 4.10. Proteasome Dysfunction and CANDLE/PRAAS Patients

While activation and upregulation of the UPS contributes essentially to skeletal muscle atrophy as discussed in the previous paragraphs, proteasome dysfunction also promotes muscle weakness in contrast to that. Muscle atrophy, myositis, lipodystrophy, arthritis, recurrent episodes of fever and skin eruptions in patients are typical features in all forms of Proteasome-Associated Autoinflammatory Syndrome (PRAAS), which is represented by sterile inflammation [392]. Several names were used by different groups to describe this disease spectrum [393]. The first direct linkage between impaired proteasome activity and chronic inflammation was discovered by Agarwal and colleagues in patients suffering from joint contractures, muscle atrophy, microcytic anemia and panniculitis-induced lipodystrophy, and they called this disease JMP (Joint contractures-muscular atrophy-microcytic anemia-panniculitis-associated lipodystrophy syndrome) syndrome. They found a homozygous missense mutation in the *PSMB8* gene encoding the immunoproteasome catalytic subunit β5i/LMP7 and detected highly increased serum levels of IL-6 and IFN-γ in the affected patients [394]. Further mutations in the same gene were then found in patients diagnosed with Japanese autoinflammatory syndrome with lipodystrophy (JASL) [395], Nakajo-Nishimura syndrome (NNS) [396], and Chronic atypical neutrophilic dermatosis with lipodystrophy and elevated temperature (CANDLE) [397] who were suffering from very similar symptoms which lead to the hypothesis that defective immunoproteasomes are the cause of the disorders. A few years later, this assumption was proven wrong when Brehm et al. described mutations in four additional proteasome genes (*PSMA3*, *PSMB4*, *PSMB9* and *POMP*) associated with PRAAS, which manifest as well in decreased proteasome activity and strong type I IFN signature. Therefore, they suggested that a global proteasome dysfunction is responsible for the chronic activation of a type I IFN response since all patients displayed decreased expression or hindered proteasome assembly and accumulation of ubiquitinated proteins [65]. Furthermore, subjects with alterations in the proteasome chaperone *PSMG2* and the remaining immunoproteasome subunit *PSMB10* (β2i/MECL-1) also developed PRAAS symptoms [398,399]. Another form of PRAAS, namely POMP-related autoinflammation and immune dysregulation disease (PRAID), was described in patients with *POMP* frameshift variants that escape nonsense-mediated decay (NMD) and interfere with proper proteasome assembly. They exhibited a severe neonatal-onset phenotype with pronounced ER stress, activation of the unfolded protein response and type I IFN response [400]. By now, it is clear that not all discovered proteasome loss-of-function mutations cause severe autoinflammation. Genomic alterations in genes of 19S subunits (*PSMD12* [401], *PSMC3* [402]) or in *PSMB1* encoding β6 [403] are predominantly characterized by neurodevelopmental disorders and do not present with pronounced autoinflammation [47,392]. In addition to type I IFN, PRAAS patients present with moderate-to-strongly increased serum levels of IL-6 [393,404]. Moreover, activation of p38 MAPK was shown in subjects with a mutated version of *PSMB8* due to reduced proteasome activity, which might further contribute to IL-6 elevation [396]. IFN-γ was only elevated in JMP and JASL syndrome, while TNF levels were increased in NNS [393]. The treatment options for PRAAS patients are limited. Many of them do not respond well to biologic disease-modifying antirheumatic drugs that target proinflammatory cytokines [65,405]. The application of baricitinib, an orally available inhibitor of JAK1 and JAK2, which decreases the phosphorylation of STAT1 and blocks type I IFN signaling, has a very promising outcome and is well tolerated in patients with interferonopathies. The treatment normalized serum levels of inflammatory markers and showed significant clinical improvement [393,405]. Two recently published studies described successful treatment of PRAAS patients by hematopoietic stem cell transplant (HSCT), providing strong evidence that immunodeficiency and autoinflammation can be traced back to defective proteasomes in hematopoietic cells [406,407].

It is still under debate what molecular mechanisms are responsible for type I IFN production following proteasome dysfunction in PRAAS patients. We and others hypothesized an involvement of increased ER stress and activation of the unfolded protein response (UPR), since it is well established that proteasome impairment affects the ERAD pathway and leads in turn to the accumulation of damaged and misfolded proteins at the ER [400,408,409]. There are various pathways that may induce sterile inflammation in response to persistent ER stress such as mechanisms including the ER-membrane sensors Activating Transcription Factor 6 (ATF6), protein kinase RNA-like endoplasmic reticulum kinase (PERK) and inositol-requiring enzyme 1-alpha (IRE1-α) as well as crosstalk with the integrated stress response (ISR) and mTORC1 activity [410,411,412].

To date, it was not formally clarified what mechanism exactly triggers the pronounced muscle atrophy in extremities of PRAAS patients. Whether or not type I IFNs play a direct role in proteasome dysfunction-induced muscle wasting remains to be determined. One can speculate that the chronic inflammation, possibly also in the muscle, and increased cytokine levels in the serum induce the severe loss of muscle mass via the previously discussed mechanisms. In addition to that, cytokines induce radical production, therefore increasing the burden of oxidant-damaged proteins and leading to more cellular stress, which cannot be counteracted by upregulation of proteasomal activity. This in turn reinforces the type I IFN response and closes the inflammation circle [413].

## 5. Conclusions

ICUAW is a devastating sequela for critically ill septic patients and inflammatory cytokines, and acute phase response proteins are involved in its pathogenesis. While the contribution of E3 ubiquitin ligases, especially atrogin-1/MAFbx and MuRF1, received much attention, the role of the proteasome in ICUAW and particular inflammation-induced skeletal muscle atrophy is less well defined. However, data from biopsy specimens of critically ill human patients, PRAAS patients, animal models of sepsis and toxic shock as well as proteasome inhibitor studies unequivocally suggest a role of the proteasome itself in ICUAW. Further studies are needed to decipher the differential role of the proteasome isoforms in muscle in ICUAW. For example, it would be interesting to know if the composition of the muscular proteasomes changes during sepsis; whether this affects their substrate specificity and which substrates are then degraded; which upstream regulators are involved in this process; to what extent this affects muscle wasting; and whether this is related to fiber types and certain muscle groups. Much of our knowledge is based on short-term animal models ranging from a few hours until four to seven days after the induction of sepsis or toxic shock. During this time frame, many septic patients have not even seen a physician. The dynamics of sepsis and inflammation as well as possible long-term side effects suggest that there is a window of opportunity to administer drugs, but this has not been sufficiently addressed so far. Proteasome dysfunction in PRAAS patients leads to muscle wasting indicating the requirement of tightly controlled and balanced protein homeostasis rather than general proteasome inhibition. If this holds true, biological or small molecules interfering with inflammatory signaling represent promising treatment options also for ICUAW patients. A new way to rebalance protein homeostasis and to control muscle wasting lies in inhibitors of E3 ubiquitin ligases involved muscle protein breakdown. It is currently uncertain if this leads to a preservation of muscle mass and function, as these are, together with survival, the most important outcomes for patients. Much emphasis has been put on molecular mechanisms and treatment options to prevent or treat ICUAW in human patients, animal models and cell culture with exciting results. Now is the time for translational approaches.
